# A Randomized, Double-Blind, Placebo-Controlled Trial on Clinical Efficacy of Topical Agents in Reducing Pain and Frequency of Recurrent Aphthous Ulcers

**DOI:** 10.2174/1745017901814010700

**Published:** 2018-09-28

**Authors:** Renu Sharma, Shambulingappa Pallagatti, Amit Aggarwal, Soheyl Sheikh, Ravinder Singh, Deepak Gupta

**Affiliations:** 1 Department of Oral Medicine and Radiology, Desh Bhagat Dental College and Hospital, Mandi Gobindgarh, Punjab- 147301, India; 2 Department of Oral Medicine and Radiology, Maharishi Markandeshwar College of Dental Science and Research, Mullana, Ambala, India

**Keywords:** Recurrent aphthous ulcer, Topical treatment, Randomized, Double-blind, Oral mucosa, Placebo-controlled trial

## Abstract

**Introduction:**

Recurrent Aphthous Ulcers (RAU) is one of the most common oral ulcerative Disease of the oral mucosa with high recurrence rate. Standard topical treatment options provide symptomatic relief with few have been found to be effective in treating or relieving the symptoms.

**Aim:**

The study aimed to evaluate the clinical efficacy of various topical agents in order to find the better treatment modality so as to decrease the number, size, exudate level and discomfort associated with pain with RAU.

**Materials and methods:**

The patients diagnosed with minor recurrent aphthous ulcers fulfilling the inclusion and exclusion criteria were enrolled. All the baseline parameters were measured by the principal investigator. The treatment modality was assigned by generating a randomization list by computer software, double-blinded in consecutively numbered sealed envelopes. The topical treatment modalities that were included: 5% Amlexanox, 0.1% Triamcinolone Acetonide, 20% Benzocaine gel, 100 mg Doxycycline hyclatemixed with denture adhesive and normal saline (20:2:1); The study was placebo controlled in which placebo gel 10 gm was used. The size, no of ulcers, pain, erythema and exudate level were measured by the principal investigator at days 1, 4, 8 and 10. All quantitative variables were estimated using measures of central tendency (mean, median) and measures of dispersion (standard deviation). Qualitative or categorical variables were described as frequencies or proportions. Proportions were compared using Post Hoc Test and N Par Tests. Effectiveness was checked using *p*-value (< 0.005).

**Results:**

It was observed that 0.1% Triamcinolone Acetonide and 5% Amlexanox proven to be more efficacious in the reduction of size, Number, Pain, Erythema and Exudate Levels at day 8, (*p* = .000*) and at day 10 (*p* =. 000*) as compared to single application of 100 mg Doxycycline Hyclate, 20% Benzocaine gel and the placebo, which was statistically significant. VAS scale was significant for 100 mg Doxycycline Hyclate and 20% Benzocaine gel.

**Conclusion:**

The selected topical treatment modality can deliver cheap, effective and safe drug therapy which benefits the patient in refining their regular activities and everyday events of life.

## 1 INTRODUCTION

Recurrent Aphthous Ulcers (RAU) is one of the most common oral ulcerative diseases of the oral mucosa with high recurrence rate [1, 2]. Although many precipitating factors have been identified, the cause as yet remains unknown. Etiological factors such as local trauma, immunodeficiency, hereditary influences, allergic agents, nutritional deficiency, hormonal imbalances in women, physical or psychic stress, chemical irritants and infective agents have been proposed [2-4].

Recurrent aphthous ulceration has three different variants minor aphthous ulcers, major aphthous ulcers and herpetiform ulcers [3, 4]. Diagnosis of RAU is based on history, clinical manifestations, and histopathology. Natah **et al**., in 2004 [5] proposed the diagnostic criteria for minor RAU.

Management of minor aphthous may be usefully divided into three phases, these include (1) Symptomatic and supportive treatment (2) Specific treatment; and (3) Preventive treatment [6]. Standard topical treatment options that provide symptomatic relief include analgesics, anaesthetics, antiseptics, anti-inflammatory agents, steroids, sucralfate, tetracycline and silver nitrate [7].

Out of all the topical applications, few have been found to be effective in treating or relieving the symptoms. The main topical therapies which have been found to be efficacious in treatment of RAU are 5% Amlexanox (inhibits the synthesis and release of inflammatory mediators, including leukotrienes and histamine, from mast cells, neutrophils, and mononuclear cells); 0.1% Triamcinolone Acetonide (inhibits the inflammatory reaction by blocking effect of the T lymphocyte-epithelial cell interaction); 20% Benzocaine (ethyl aminobenzoate, local anaesthetic to reduce pain by inhibiting inflammatory reaction) and Doxycycline Hyclate (inhibitor of matrix metalloproteinases which play a role in tissue destructive events) [8]. Properties such as the inhibition of prostagland in production, inhibition in the formation and release of histamine and leukotriene’s from mast cells, neutrophils and mononuclear cells, leukocyte suppression, inhibition of collagenase and gelatinase and anti-inflammatory and anti-allergic properties [8] are requisites for topical medication for RAU and no single modality has been successful.

The present study was aimed to evaluate the clinical efficacy of various topical agents in order to find the better treatment modality so as to decrease the number, size, exudate level and discomfort associated with pain with RAU.

## 2 MATERIALS AND METHODS

### 2.1 Patient Selection

The prospective clinical study was conducted from July 2014 to June 2015. The sample consisted of patients with known history of recurrent minor ulcers and with single or multiple ulcers with size less than 10 mm in diameter and duration less than 72 hours. The recurrent minor ulcers are characterized by size varying from 5 to 10 mm, seen in the non-keratinized mucosal surfaces and healing within 10-14 days without scarring.

#### 2.1.1 Inclusion Criteria

History of recurrent minor ulcers with at least 3 attacks in the past three years and at the different focal site with size less than 1 cm.

#### 2.1.2 Exclusion Criteria

Patients with a history of any systemic disease or syndromes resulting in ulcers; systemic or topical drug therapy in past six months or any concurrent mucosal lesions were excluded. Smokers, pregnant and lactating mothers were also excluded.

### 2.2 Methodology

The study was conducted after obtaining the Ethical approval from the Institutional Research Ethics Committee *MMU/RP430*. The patients diagnosed with minor recurrent aphthous ulcers who gave consent to participate in the study were enrolled fulfilling the inclusion and exclusion criteria and all the baseline parameters were measured by the principal investigator. The treatment modality was assigned by generating a randomization list by computer software (*random allocation software 2.0*), blinded in consecutively numbered sealed envelopes by another clinician who had the key, blinded both to the principal investigator and patients. A “double blinded” approach is when both physician and patients don’t know which is the treatment received. In these cases, at least the personnel dedicated to the evaluation of the response to the treatment should do not have information regarding group allocation. This is done to avoid subjectivity in outcome assessing and to permit the reliability and the objectivity of the results. To accurately evaluate the effectiveness, the treatment modalities that were included; were in measured tubes numbering 10 each: of 5% Amlexanox (Trade name-Lexanox 5 gm), 0.1% Triamcinolone acetonide (Trade name-Kenacort 5 gm), 20% Benzocaine gel (Trade name-Mucopain 15 gm); 100 mg Doxycycline Hyclate, (Trade name-DOXT-S) mixed with denture adhesive and normal saline (20:2:1); and placebo gel-10 gm (Placebo gel contained a mixture of mineral oil, gelatin, pectin, carboxymethylcellulose sodium, methylcellulose sodium, glycerol and white petroleum percipients except the active ingredient); making a total of 50 treatment tubes. All the topical ointments were enclosed in identical sealed covers, were computer coded and allotted by the second clinician having the key for the treatment options. After measuring the baseline parameters by the principal investigator, the subject was allotted the coded tubes randomly selected by computer programme dividing the subjects 5 groups without discriminating on the basis of age and gender factors by another clinician blinded to the selected parameters.

### 2.3 Measurements

After enrolment, the size, no of ulcers, pain, erythema and exudate level were measured by the principal investigator at days 1, 4, 8 and 10. Ulcer size was measured using a sterile William’s calibrated dental probe with millimeter marking and the longest diameter was used as measurement and the pain was evaluated by the subjects based on Visual Analogue Scale before drug application and at following days.

#### 2.3.1 Classification of Erythema and Exudation Levels

### 2.4 Procedure

Patients were instructed to squeeze out approximately ¼ inch (0.5 cm) of the paste from the tube on a clean fingertip and apply to the site of the ulcer without spreading it to the surrounding area. They were then instructed to apply the drug 4 times a day, preferably following oral hygiene after breakfast, lunch, dinner and at bedtime. To check for reliability, all patients were asked to apply in front of the second clinician for the first time and instructed to maintain a regular chart of application.

Single application of Doxycycline Hyclate**:** The single application of Doxycycline Hyclate was administered by the other clinician on the first visit of the patient in that group. The powdered doxycycline hyclate was mixed with denture adhesive and normal saline with 20:2:1 ratio and was directly placed over the ulcer using a plastic instrument.

The patients were observed for 30 minutes for any possible signs of acute hypersensitivity reactions and were instructed to avoid drinking or eating for 30 minutes after each administration of the drug. And they were further recalled on 4^th^, 8^th^ and 10^th^ day in morning following the treatment procedure for evaluating all the parameters.

### 2.5 Statistical Analysis

The statistical analysis was carried out using statistical package for social sciences (IBM *SPSS* Inc., Chicago Version 22.0for window). All quantitative variables were estimated using measures of central tendency (mean, median) and measures of dispersion (standard deviation). Qualitative or categorical variables were described as frequencies or proportions. Proportions were compared using Post Hoc Test and N Par Tests.

## 3 RESULTS

**Fig. (1):**
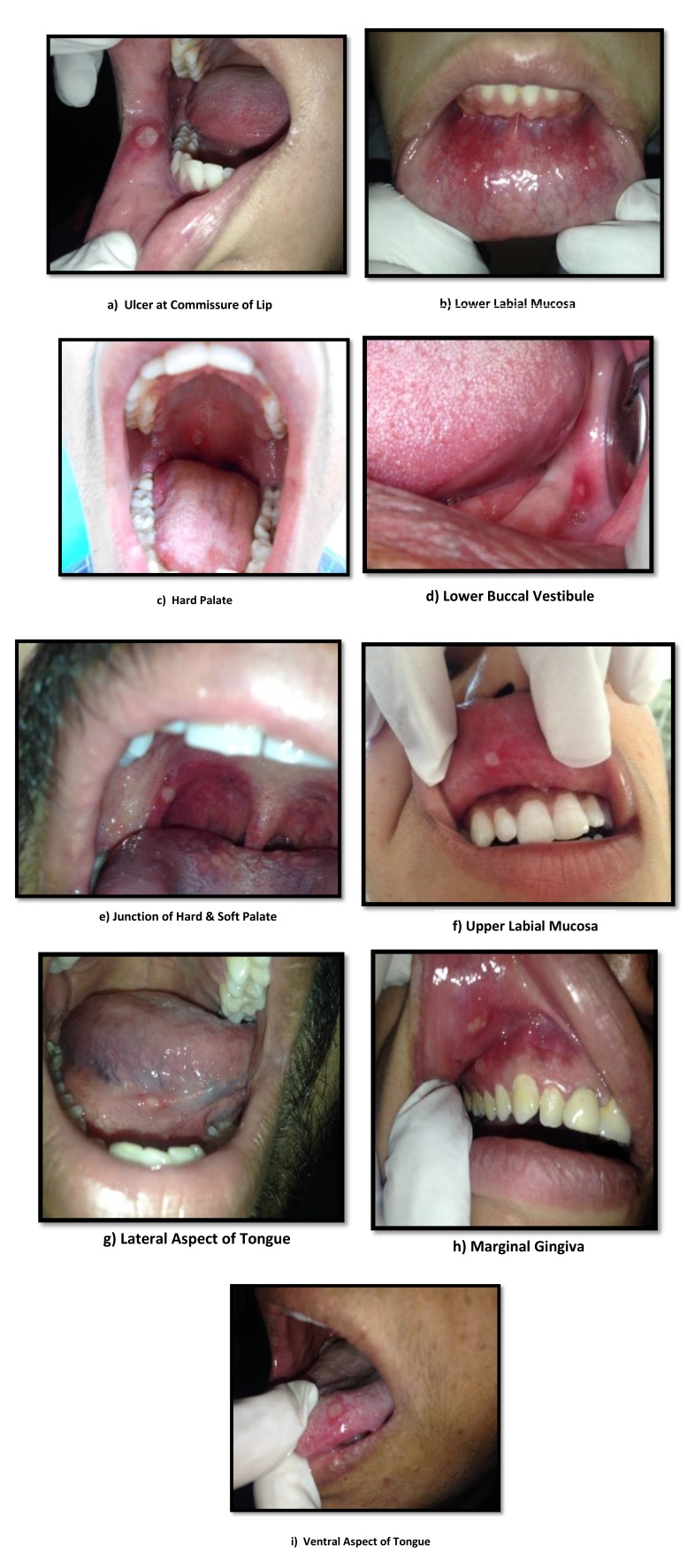
Different sites involved in recurrent aphthous ulcers.

**Fig. (2):**
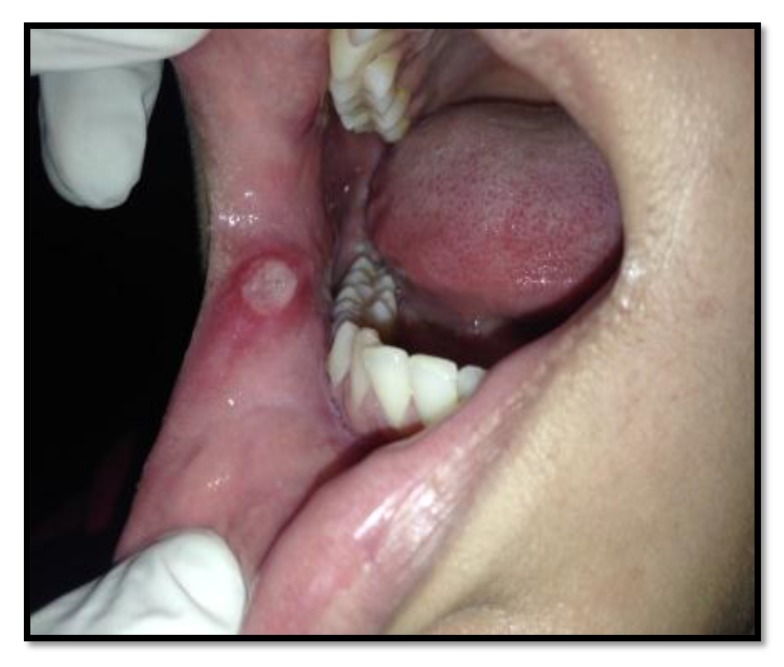
Recurrent aphthous ulcer day 1.

Out of the 50 patients assessed, 1 patient did not complete the study period giving an overall response rate of 98%. The mean age of the subjects was 26.5 years. The male to female ratio was 1:1.This study has shown a reduction in number, size, erythema, exudate, pain associated with ulcers (Figs. **1a**-**i**, Fig. **2**) and also discomfort on chewing food with no side-effects attributed to the drugs. None of the patients reported any allergy to any therapy; minor taste alterations were there which were negligible with no complaints.

**Fig. (3):**
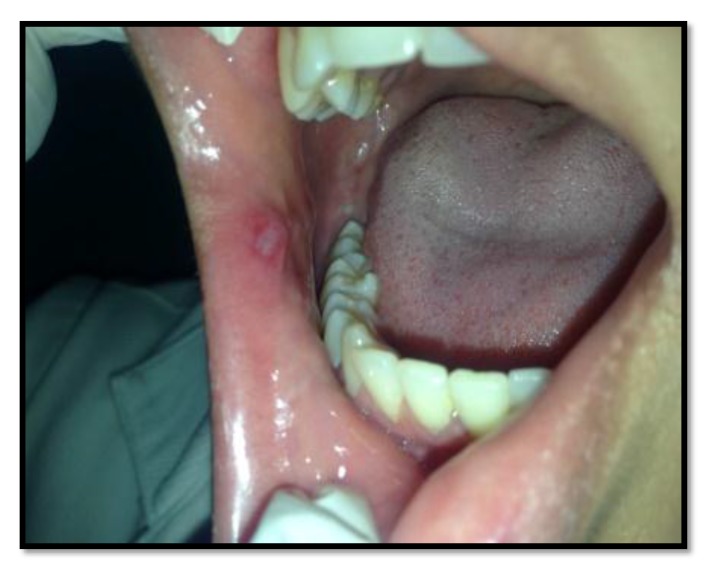
Recurrent aphthous ulcer Day 4.

**Fig. (4):**
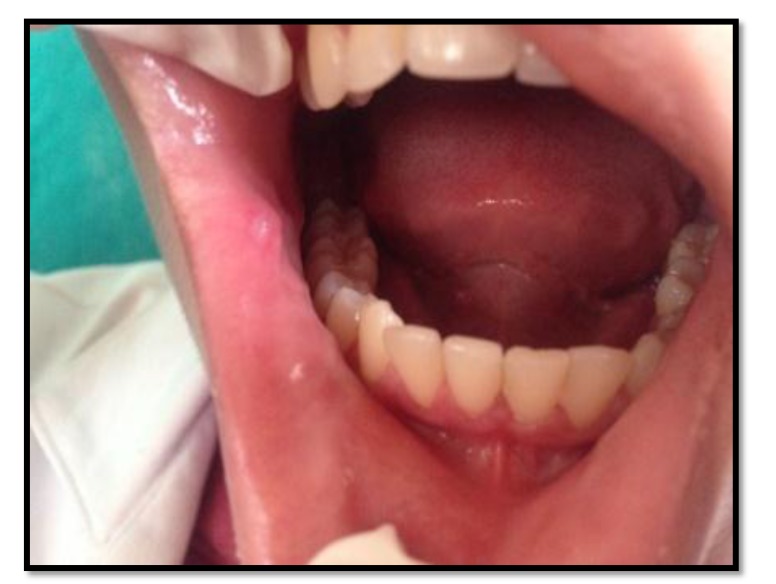
Recurrent aphthous ulcer Day 8.

**Fig. (5):**
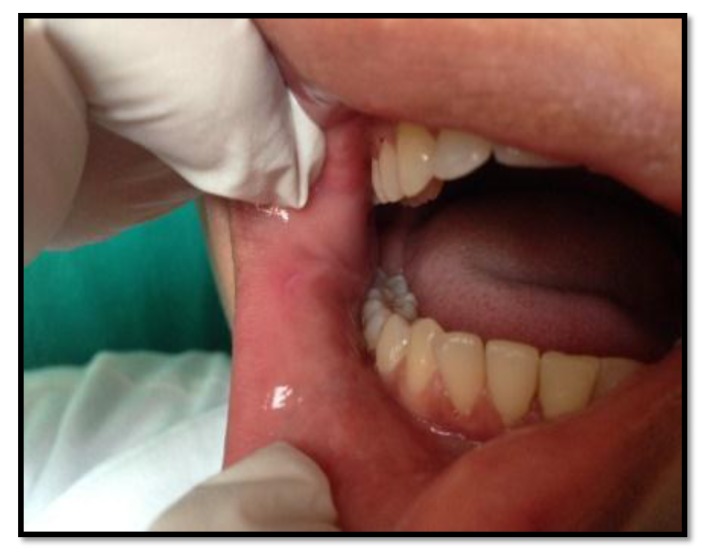
Recurrent aphthous ulcer day 10.

**Graph (1):**
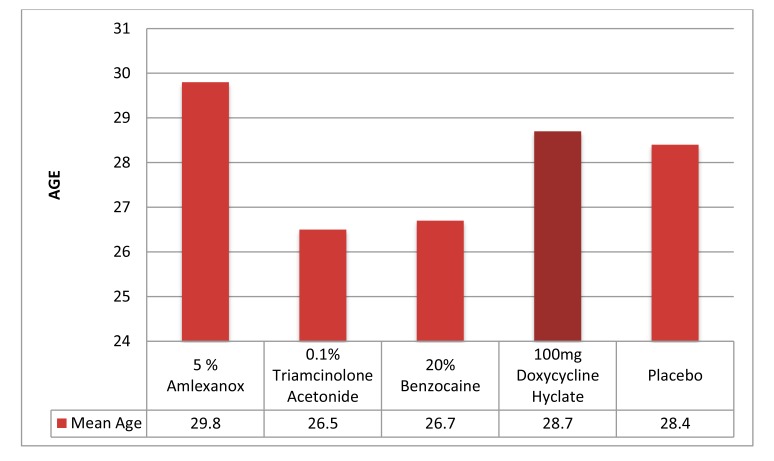
Age wise distribution of subjects.

**Graph (2):**
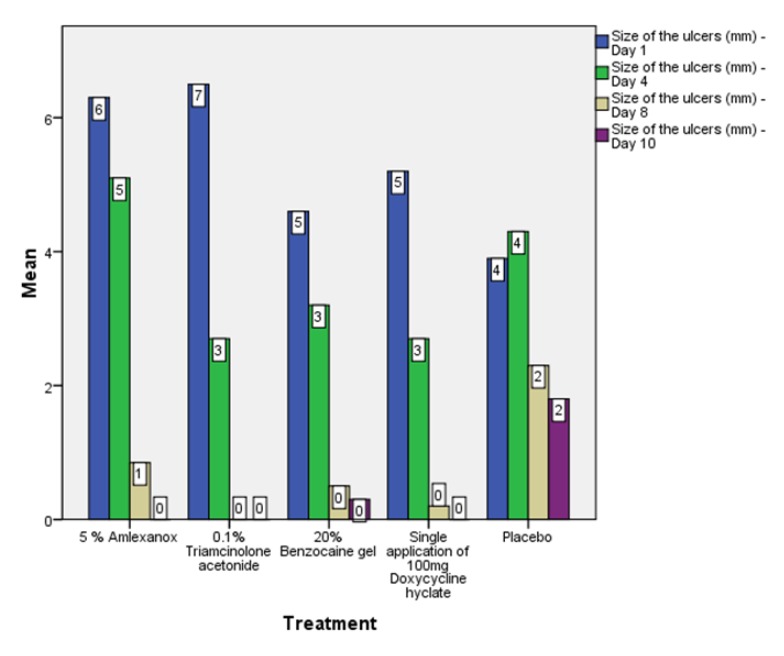
Comparison of treatment modalities for the size of the ulcers.

**Graph (3):**
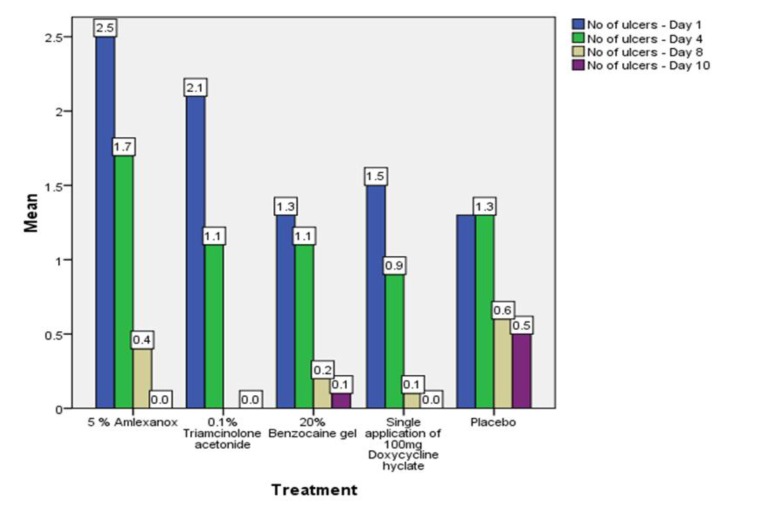
Comparison of treatment modalities for the number of the ulcers.

**Graph (4):**
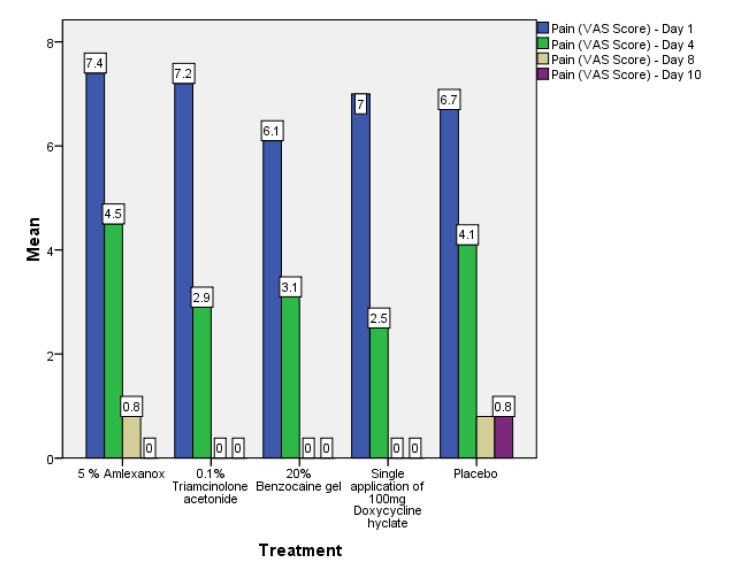
Comparison of treatment modalities for the pain (VAS score).

**Graph (5):**
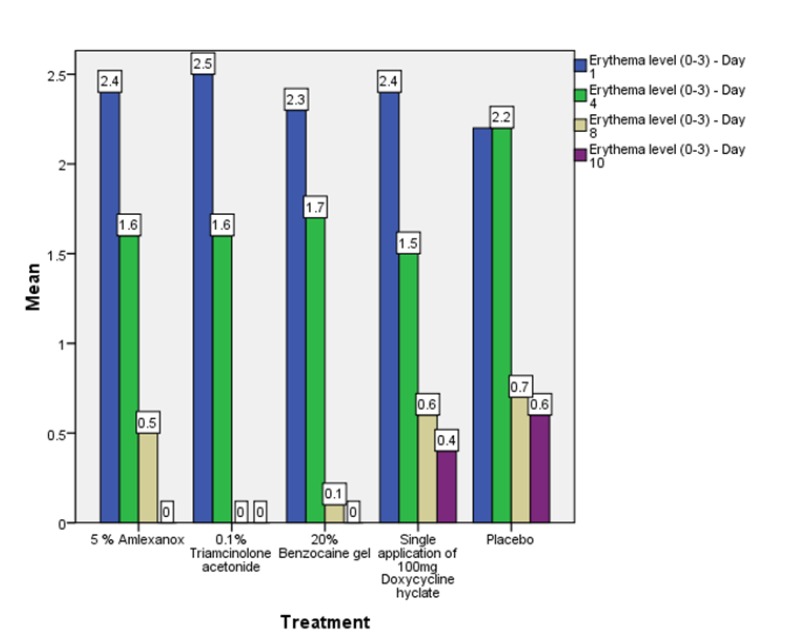
Comparison of treatment modalities for the erythema level.

On the first recall visit *i.e.* on day 4 (Fig. **3**), 0.1% Triamcinolone Acetonide and 100 mg doxycycline hyclate were found to be more efficacious in reducing the size of the ulcers and pain; all modalities except placebo was effective in reducing the number of ulcers, erythema and exudate. It was observed that 0.1% Triamcinolone Acetonide and 5% Amlexanox proven to be more efficacious in reduction of size, number, pain, erythema and exudate Levels at day 8 (Fig. **4**), (*p* = .000*) and at day 10 (*p* = .000*) as compared to single application of 100 mg Doxycycline Hyclate, 20% Benzocaine gel and the placebo, which was statistically significant (Table **3**). The symptomatic relief in the Pain (VAS) was found to be significant at the day 10 (Fig. **5**) in subjects treated with single application of 100 mg Doxycycline Hyclate and 20% Benzocaine gel (*p* = .001) (Tables **1**-**5**) (Graphs **1**-**5**).

## 4 DISCUSSION

Recurrent aphthous stomatitis or aphthous ulcers are more common in younger adults. There are several causes that have been explained for ulcer formation but no single cause is definitive. The cause is still non-specific. There are multiple factors which may be acting together in a complex manner in initiating the formation of ulcer unlike a single etiological factor. This means a combination of host and environmental factors are essential not only for triggering the ulcer but also for an increase in size. The severity of etiological factors to which an individual is exposed would decide the type of ulcer [2, 9].

It was observed that recurrent aphthous ulcers are equally prevalent in both the genders. The mean age of the subjects was 26.5 years which was in accordance with the patients taken in previous studies and the peak age of occurrence is second decade of the life. It involves whole of the oral mucosa but most commonly it was seen on the lower labial mucosa. Recurrent aphthous stomatitis or aphthous ulcers are more common in younger adults. Scully **et al**. [10] stated that in about 80% of patients with RAU, the condition develops before 30 years of age and if onset is there in later stages of life it suggests definable predisposing factors leading to more complex form of recurrent aphthous ulceration [10]. RAU is a significant deterrent to productivity outcome of the individuals especially in younger age groups, which is a loss to the society as a whole. The painful ulceration may present significant problems to the patient; difficulty eating, speaking and swallowing can severely affect a patient's quality of life.

The lower labial mucosa was the most affected site followed by upper labial mucosa and tongue and least affected was lingual surface. Other studies show no site predilection, though it is most commonly seen in the non-keratinized mucosal surfaces like labial mucosa, buccal mucosa, and floor of the mouth.

The patients with 5% Amlexanox reported reduced number of ulcers on comparison to pre-treatment period (*p* =.000*). There was reduction in the size, pain and erythema on comparison between the groups, at the day 4 when compared to 20% Benzocaine and the Placebo group. Meng **et al**., [11] conducted a placebo controlled, randomized, blinded, multicentre clinical trial comprising of 213 subjects and demonstrated that the 5% Amlexanox oral adhesive pellicles could not only reduce the ulcer size but also resolve the pain of the patients during the RAU treatment lasting for 5 days. He indicated that Amlexanox oral adhesive pellicles are as effective and safe as Amlexanox oral adhesive tablets in the treatment of minor RAU. The clinical efficacy of 5% Amlexanoxhas also been advocated in studies by Bhat S **et al**., [12] and Murray **et al**., [13]. The present study had observed the potential of this drug in reducing the size of the ulcer, pain, erythema and exudation. Amlexanox oral paste was well tolerated during the study period. Amlexanox inhibits the synthesis and release of inflammatory mediators, including leukotrienes and histamine, from mast cells, neutrophils, and mononuclear cells. Amlexanox also acts as a leukotriene D4 antagonist and a phosphodiesterase inhibitor. Amlexanox decreases the time ulcers take to heal as well as the pain associated with the ulcers [14].

Triamcinolone Acetonide (0.1%) was observed to be effective in the treatment of RAU as also seen by studies conducted by Miles D **et al**., [14], MM Fanil**et al**., [15] but in that study comparison was made between Phenytoin Syrup and Triamcinolone Acetonide Ointment. There was not any study to compare the effect of triamcinolone with other topical medications. As seen in one of the studies, it has shown the largest margin of efficacy in patients with severe RAU with a (*p* =.000*). Triamcinolone acetonide is a fluoride synthetic corticosteroid. Topical Steroids act by inhibiting the inflammatory reaction by blocking the effect of the T lymphocyte-epithelial cell interaction. Since the concentration of sensitized lymphocytes occurs before and during the early stages of oral ulceration, it follows that the drugs exert their maximum effect at this time and decrease the duration of the aphthosis. But Long-term use of local steroids may also predispose to local candida infection [5].

Few studies have explored the therapeutic effectiveness of doxycycline hyclate in RAS. In a study conducted by Ylikontiola **et al**., [16] doxycycline was used in a powdered form as in our study but the adhesive used to retain the medicament was isobutyl cyanoacrylate. Another study used sub-antimicrobial dose of doxycycline 20 mg to prevent the recurrence of RAS. The present study using powdered doxycycline in a denture adhesive base is one of the few studies of this kind attempted for the pain palliation in RAS patient. The mechanism of action of doxycycline hyclate is through inhibition of interstitial collagenases (matrix metalloproteinases), inhibition of prostaglandin production, leukocyte suppression which play a major role in tissue destructive events in RAU and anti-microbial properties [17]. This was in accordance with the study conducted by Muzio **et al**., [18] and Vijyabala GS **et al**. [17] in which adhesive denture paste was administered to the patients with RAS and a good retention of the medicament to the mucosa was achieved.

Another treatment modality advocated was placebo and we had observed some healing effects in the placebo group as well. The patient which was lost due to non-compliance belonged to this group. There are three possible reasons to explain the healing effects of placebo on RAU. First, the glycerol and petroleum form a protective film to cover the ulcer and therefore, produce some remedial effect. Second, minor aphthous ulcers are a self-limiting disease, which can get reprieve without any treatment by day 8^th^ or day 10^th^. Finally, stress was known as one of the triggering factors, and subjects of the placebo group might have some psychological therapeutic benefit [19].

No patient reported new ulcers during the study period. On the basis of efficacy, cost, and safety, topical medications remain the first choice of treatment for patients with RAU. In this study we had observed 5% Amlexanox and 0.1% Triamcinolone Acetonide as the most effective in terms of cost-effectiveness and as well as symptomatic relief of all the topical agents used so far for the treatment of recurrent aphthous ulcers followed by Single application of Doxycycline Hyclate and 20% Benzocaine Gel.

Low-level laser therapy (LLLT), also known as “soft laser therapy,” “laser phototherapy” (LPT), and “cold laser therapy” has been advocated in recent years as an alternative treatment for RAUs by modulating inflammatory responses with a reduction in oedema and pain and cellular biostimulation, but taking into account the fact that there are few trials published in the literature concerning LLLT for the treatment of minor RAUs, it is not possible to dictate that this specific protocol should be used by clinicians [20] .

As recurrent aphthous ulcers have a self-remission period of a week to ten days, this was seen as one of the shortcoming of this study. Another disadvantage was that the topical application is totally patient dependent and irregular use of the drug might be possible. This can hinder the results and comparison with other treatment modalities. But the topical drug delivery helps not only in reducing the size of ulcers but also alleviates the comfort level with which patient carries on with day to day activities and ease in chewing and swallowing food [21].

Topical therapy alone does not decrease the formation of new lesions and may not be adequate treatment for patients with major RAU or patients who experience frequent episodes of multiple minor RAU [22-24]. Moreover, topical medications are washed away from the target area so therefore, it would be better to use different kinds of adhesive vehicles in combination with the drugs. Another limitation was in the allocation of random treatment modalities. As we have made an equal number of treatment tubes, the allocation to the last patient could not be randomized. The randomized studies therefore usually have an unequal number of treatment modules with patients. This study was conducted on a small scale for short period of time. Large-scale trial for a long period of time with extended follow-up is necessary.

## CONCLUSION

It can be concluded that 0.1% Triamcinolone Acetonide and 5% Amlexanox were better topical treatment modalities compared to other topical therapies in patients suffering from Recurrent Aphthous Ulcers. Although the results of the study are encouraging and showed reduction in all the parameters by topical treatment modalities, it has shown tremendous scope for further research in the management of RAU. However, the results of this study confirmed that with topical modality only, we can deliver cheap, effective and safe drug therapy which had benefit the patient in refining their regular activities and everyday events of life.

## Figures and Tables

**Graph (6):**
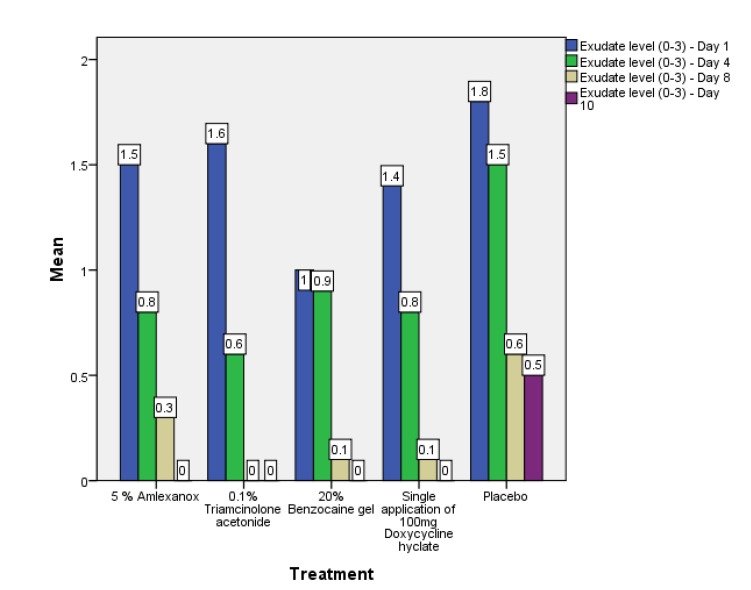
Comparison of treatment modalities for the exudate level.

**Table:** 

**Classification**	**Erythema Level**	**Exudation Level**
0	No erythema	No exudation
1	Light red/pink	Light exudation
2	Red but not dark in colour	Moderate exudation
3	Very red, dark in colour	Heavy exudation, with pseudo membrane

**Table 1:** Comparison among topical modalities for size of ulcers (mm).

[1] Days	Group	Mean (mm)	Mean Difference & Std. Deviation	*P* value
Size of ulcers Day 1	5% Amlexanox	6.30	–	
	0.1%Triamcinolone acetonide	6.50	0.20±2.24	1.000
	20% Benzocaine gel	4.60	1.70±1.44	.918
	100 mg Doxycycline hyclate	5.20	1.10±1.42	.994
	Placebo	3.90	2.40±1.41	.620
Size of ulcers Day 4	5% Amlexanox	5.10	–	
	0.1%Triamcinolone acetonide	2.70	2.40±1.79	.847
	20% Benzocaine gel	3.20	1.90±1.70	.939
	100 mg Doxycycline hyclate	2.70	2.40±1.60	.757
	Placebo	4.30	.80±1.51	1.000
Size of ulcers Day 8	5% Amlexanox	.850	–	
	0.1%Triamcinolone acetonide	.000	.85±.51	.661
	20% Benzocaine gel	.500	.35±.71	1.000
	100 mg Doxycycline hyclate	.200	.65±.55	.914
	Placebo	2.300	1.45±1.02	.801
Size of ulcers Day 10	5% Amlexanox	.00	–	
	0.1%Triamcinolone acetonide	.00	.00±.00	.000*
	20% Benzocaine gel	.30	0.30±0.30	.961
	100 mg Doxycycline hyclate	.00	0.00±0.00	.000*
	Placebo	1.80	1.80±.80	.330

**Table 2:** Comparison among topical modalities for number of ulcers.

Days	Group	Mean	Mean Difference & Std. Deviation	*P* value
No of ulcers Day 1	5% Amlexanox	2.50	–	
	0.1%Triamcinolone acetonide	2.10	.40±.89	1.000
	20% Benzocaine gel	1.30	1.20±.60	.432
	100 mg Doxycycline hyclate	1.50	1.00±.60	.653
	Placebo	1.30	1.20±.56	.367
No of ulcers Day 4	5% Amlexanox	1.70	–	
	0.1%Triamcinolone acetonide	1.10	.60±.67	.986
	20% Benzocaine gel	1.10	.60±.54	.946
	100 mg Doxycycline hyclate	.90	.80±.52	.741
	Placebo	1.30	.40±.47	.989
No of ulcers Day 8	5% Amlexanox	.40	–	
	0.1%Triamcinolone acetonide	.00	.40±.22	.561
	20% Benzocaine gel	.20	.20±.29	.998
	100 mg Doxycycline hyclate	.10	.30±.24	.890
	Placebo	.60	0.20±.31	.999
No of Ulcers Day 10	5% Amlexanox	.00	–	
	0.1%Triamcinolone acetonide	.00	.00±.00	.000*
	20% Benzocaine gel	.10	0.10±.10	.961
	100 mg Doxycycline hyclate	.00	.00±.00	.000*
	Placebo	.50	0.50±.22	.336

**Table 3:** Comparison among topical modalities for Pain score (VAS).

Days	Group	Mean	Mean Difference & Std. Deviation	*P* value
Pain (VAS) Day 1	5% Amlexanox	7.40	–	
	0.1%Triamcinolone acetonide	7.20	0.20±0.76	1.000
	20% Benzocaine gel	6.10	1.30±.74	.580
	100 mg Doxycycline hyclate	7.00	0.40±0.70	1.000
	Placebo	6.70	0.70±0.70	.969
Pain (VAS) Day 4	5% Amlexanox	4.50	–	
	0.1%Triamcinolone acetonide	2.90	1.60±1.35	.918
	20% Benzocaine gel	3.10	1.40±1.31	.953
	100 mg Doxycycline hyclate	2.50	2.00±1.36	.772
	Placebo	4.10	0.40±1.35	1.000
Pain (VAS) Day 8	5% Amlexanox	.80	–	
	0.1%Triamcinolone acetonide	.80	0.80±0.55	.780
	20% Benzocaine gel	.00	0.80±0.55	.780
	100 mg Doxycycline hyclate	.00	0.80±0.55	.780
	Placebo	.00	0.00±0.78	1.000
Pain(VAS) Day 10	5% Amlexanox	.00	–	
	0.1%Triamcinolone acetonide	.00	0.00±0.00	.000*
	20% Benzocaine gel	.00	0.00±0.00	.000*
	100 mg Doxycycline hyclate	.00	0.00±0.00	.000*
	Placebo	.80	0.80±0.55	.780

**Table 4:** Comparison among topical modalities for erythema levels (Grer *et al.*, scale).

Days	Group	Mean	Mean Difference & Std. Deviation	*P* value
Erythema Level Day 1	5% Amlexanox	2.40	–	
	0.1%Triamcinolone acetonide	2.50	0.10±0.23	1.000
	20% Benzocaine gel	2.30	0.10±0.30	1.000
	100 mg Doxycycline hyclate	2.40	0.00±0.23	1.000
	Placebo	2.20	0.20±0.29	.998
Erythema Level Day 4	5% Amlexanox	1.60	–	
	0.1%Triamcinolone acetonide0	1.60	0.00±0.54	1.000
	20% Benzocaine gel	1.70	0.10±0.47	1.000
	100 mg Doxycycline hyclate	1.50	0.10±0.52	1.000
	Placebo	2.20	0.60±0.44	.841
Erythema Level Day 8	5% Amlexanox	.50	–	
	0.1%Triamcinolone acetonide	.00	0.50±0.22	.336
	20% Benzocaine gel	.10	0.40±0.24	.666
	100 mg Doxycycline hyclate	.60	0.10±0.37	1.000
	Placebo	.70	0.20±0.34	.999
Erythema Level Day 10	5% Amlexanox	.00	–	
	0.1%Triamcinolone acetonide	.00	0.00±0.00	.000*
	20% Benzocaine gel	.00	0.00±0.00	.000*
	100 mg Doxycycline hyclate	.40	0.40±0.26	.748
	Placebo	.60	0.60±0.26	.330

**Table 5:** Comparison among topical modalities for exudate level (Grer *et al*., scale).

Days	Group	Mean	Mean Difference & Std. Deviation	*P* value
Exudate Level Day 1	5% Amlexanox	1.50	–	
	0.1%Triamcinolone acetonide	1.60	0.10±0.23	1.000
	20% Benzocaine gel	1.00	0.50±0.16	.114
	100 mg Doxycycline hyclate	1.40	0.10±0.27	1.000
	Placebo	1.80	0.30±0.33	.984
Exudate Level Day 4	5% Amlexanox	.80	–	
	0.1%Triamcinolone acetonide	.60	0.20±0.25	.995
	20% Benzocaine gel	.90	0.10±0.22	1.000
	100 mg Doxycycline hyclate	.80	0.00±0.28	1.000
	Placebo	1.50	0.70±0.33	.370
Exudate Level Day 8	5% Amlexanox	.30	–	
	0.1%Triamcinolone acetonide	.00	0.30±0.15	.472
	20% Benzocaine gel	.10	0.20±0.18	.944
	100 mg Doxycycline hyclate	.10	0.20±0.18	.944
	Placebo	.60	0.30±0.26	.939
Exudate Level Day 10	5% Amlexanox	.00	–	
	0.1%Triamcinolone acetonide	.00	0.00±0.00	.000*
	20% Benzocaine gel	.00	0.00±0.00	.000*
	100 mg Doxycycline hyclate	.00	0.00±0.00	.000*
	Placebo	.50	0.50±0.22	.336
